# Assessing the Value of DNA Barcodes for Molecular Phylogenetics: Effect of Increased Taxon Sampling in Lepidoptera

**DOI:** 10.1371/journal.pone.0024769

**Published:** 2011-09-09

**Authors:** John James Wilson

**Affiliations:** Department of Integrative Biology, University of Guelph, Guelph, Ontario, Canada; American University in Cairo, Egypt

## Abstract

**Background:**

A common perception is that DNA barcode datamatrices have limited phylogenetic signal due to the small number of characters available per taxon. However, another school of thought suggests that the massively increased taxon sampling afforded through the use of DNA barcodes may considerably increase the phylogenetic signal present in a datamatrix. Here I test this hypothesis using a large dataset of macrolepidopteran DNA barcodes.

**Methodology/Principal Findings:**

Taxon sampling was systematically increased in datamatrices containing macrolepidopteran DNA barcodes. Sixteen family groups were designated as concordance groups and two quantitative measures; the taxon consistency index and the taxon retention index, were used to assess any changes in phylogenetic signal as a result of the increase in taxon sampling. DNA barcodes alone, even with maximal taxon sampling (500 species per family), were not sufficient to reconstruct monophyly of families and increased taxon sampling generally increased the number of clades formed per family. However, the scores indicated a similar level of taxon retention (species from a family clustering together) in the cladograms as the number of species included in the datamatrix was increased, suggesting substantial phylogenetic signal below the ‘family’ branch.

**Conclusions/Significance:**

The development of supermatrix, supertree or constrained tree approaches could enable the exploitation of the massive taxon sampling afforded through DNA barcodes for phylogenetics, connecting the twigs resolved by barcodes to the deep branches resolved through phylogenomics.

## Introduction

An unprecedented amount of homologous DNA sequence data has been generated and made publicly available in the last few years as a result of the DNA barcoding movement [1]–[2]. DNA barcoding refers to the technique of sequencing a short fragment of the mitochondrial *cytochrome c oxidase subunit I* (COI) gene from a taxonomically unknown specimen and performing comparisons with a reference library of sequences of known species origin to establish a species-level identification [3]. While the goal of DNA barcoding is explicitly to aid species identification [4], mtDNA has frequently been used for phylogenetic inference at multiple taxonomic levels [5]–[7] prompting many researchers to contemplate the phylogenetic value of DNA barcode datasets [3], [5]–[9]. A common perception is that DNA barcode datamatrices have limited phylogenetic signal due to the presence of few ‘informative’ characters [7], [10]. However, a long-standing debate has focused on the relative benefits of adding more taxa versus more characters to a phylogenetic problem [11]–[14] with many authors concluding increased taxon sampling may be equally if not more beneficial [11]–[14]. For example, Hillis [14] suggested that given limited amount of time and money for datamatrix assembly, phylogenetic inferences could improve with the addition of taxa even if the total number of characters examined remains unchanged [12]. Increasing the phylogenetic signal in a datamatrix [3], [15] through increased taxon sampling may be particularly effective with DNA barcode datasets where hundreds or even thousands of species can be added to analyses.

### Phylogenetic analysis of DNA barcodes

The DNA barcode is a highly conserved protein-coding gene fragment that also has fast evolving (synonymous) nucleotide sites [16] providing species-level resolution required for barcoding. At deeper divergences these sites can appear highly homoplastic [17], due to the frequent occurrence of multiple, superimposed nucleotide substitutions, and may be considered uninformative or even misleading regarding taxonomic relationships [10] (i.e. low phylogenetic signal). Concern about saturation - the superimposed nucleotide substitutions masking any phylogenetic signal - seems largely to stem from the accepted wisdom that phylogeny inference using parsimony requires small amounts of evolution, or even the absence of homoplasy [17]–[18]. However, parsimony may perform well in spite of multiple substitutions at the same nucleotide position along a branch [19] and cladogram resolution and clade support generally decreases when excluding or down weighting synonymous positions [10], [17]–[18], [20]. Increasing taxon sampling can shorten branches meaning characters that are globally homoplastic can now become local synapomorphies [21]. Comprehensive taxonomic coverage could ultimately be the major factor determining phylogenetic signal in single gene datamatrices [5], [8].

### Evaluating the accuracy of a phylogenetic inference

**Figure 1:**
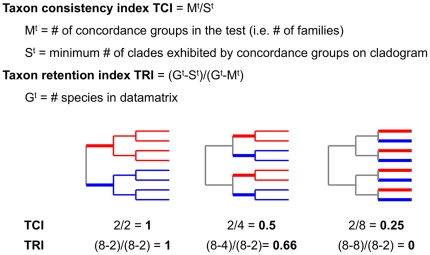
Assessing phylogenetic signal through taxonomic congruence. The equations used tocalculate the scores for two measures of phylogenetic signal through taxonomic congruence used in this study. Example calculations are shown for three cladograms each containing two concordance groups (M^t^ = 2), one red, one blue, with each group having 4 species (G^t^ = 8). In practice the scores can be calculated by coding membership in a concordance group as a character in a datamatrix and obtaining CI and RI values from PAUP.

Although the accuracy of phylogenetic inference can never be known [15], except when using simulated evolution (e.g. [22]), proxy measures are commonly used. The accuracy of a phylogenetic inference is directly related to phylogenetic signal - assessed through the ability of the datamatrix to cluster taxonomically related species together [3]. Phylogenetic signal is necessarily measured after phylogenetic analysis and can be measured a) through character congruence within the current datamatrix (the CI and RI [3]) or; b) through taxonomic congruence of the new inference with an inference produced from an independent character set. As the current taxonomic classification represents a consensus phylogenetic inference, measures of phylogenetic signal through taxonomic congruence can be formalized through the designation of concordance groups derived from taxonomy (e.g. [23]–[25], but see [26]). Using this approach phylogenetic signal has typically been assessed qualitatively, however, can be easily quantified by measuring the proportion of concordance groups recovered as monophyla [3]. An obvious weakness of this measure is that it is based on the presence/absence of a limited number of branches in a cladogram containing potentially thousands of branches; the probability of a group of species forming a clade decreases as the number of species increases. To address this weakness the taxon consistency index (TCI) [3] gives a partial score for the presence of other branches indicative of phylogenetic signal e.g. if the taxon forms only two clades (Figure 1) and the taxon retention index (TRI) [3] scales for the number of species (Figure 1). These may be more informative measures of the strength of the phylogenetic signal [3].

### Lepidoptera as a test group

The order Lepidoptera, despite apparently abundant amounts of visual variation and species diversity, exhibits a morphological homogeneity [27] that has provided only a limited number of useful taxonomic characters. This has led to widespread use of molecules for inferring taxonomic relationships [10], [17], [28]–[31]. However, previous attempts at assessing the effects of increased taxon sampling have not been particularly thorough, for example, Mitchell et al. [23] increased species coverage from 0.11% to 0.17% in the superfamily under investigation (Noctuoidea [32]) for a two gene dataset. The All Lepidoptera Barcode of Life campaign (http://www.lepbarcoding.org) aims to sequence 650bp of COI from all 160,000 lepidopteran species [33]–[34] eventually enabling comprehensive coverage of species diversity albeit for a single gene. Wilson [3] found that DNA barcode datamatrices contained strong phylogenetic signal at the genus level but that this reduced at deeper levels of the taxonomic hierarchy. However, the taxon sample size was small (977 species from 20 families) and researchers have reported observing a general phenomenon of species from the same families producing fewer clades (i.e. families approaching monophyly) on DNA barcode trees as taxon sampling within a family has increased (e.g. [33]).

While major advances have been made recently in elucidating the lepidopteran phylogeny [30]–[31], classification takes time to catch up [35]. The families, however, occupy a special place in the lepidopteran taxonomic hierarchy, and in contrast to the groups at most other taxonomic ranks (tribes and subfamilies [29], [36] and superfamilies [30]–[31]) have generally been well accepted as monophyla [28], [30]–[31]. Consequently families were used as the concordance groups in this study.

**Figure 2:**
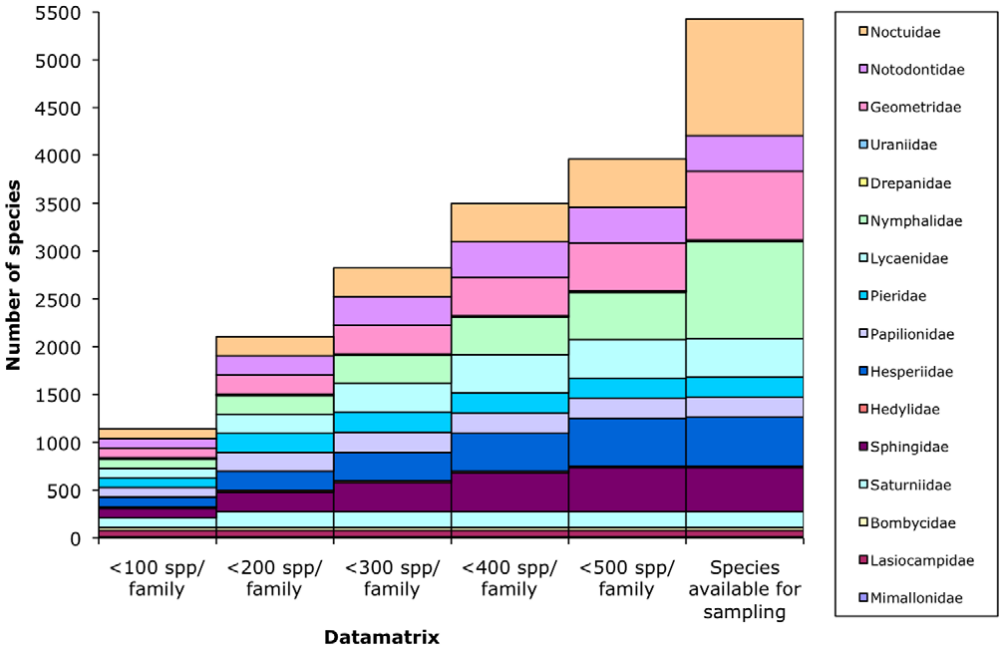
Taxon sampling schemes used to assess the effect of increased taxon sampling on phylogenetic signal in DNA barcode datamatrices. Arctiinae, Lymantrinae and Riodininae species were not included in the sample given current uncertainty over their family level status [28], [52]–[53]. See also Table S2.

In this study I test the hypothesis that increased taxon sampling will increase phylogenetic signal in a DNA barcode datamatrix. New blocks of taxa, comprising of macrolepidopteran species, were added sequentially to datamatrices containing only DNA barcodes as the character set (Figure 2). Any improvements in the accuracy of the phylogenetic inference were assessed based on two quantitative measures of phylogenetic signal (the TCI and TRI) [3] derived from the recovery of macrolepidopteran families as monophyla.

## Results and Discussion

### Effect of increased taxon sampling

**Figure 3:**
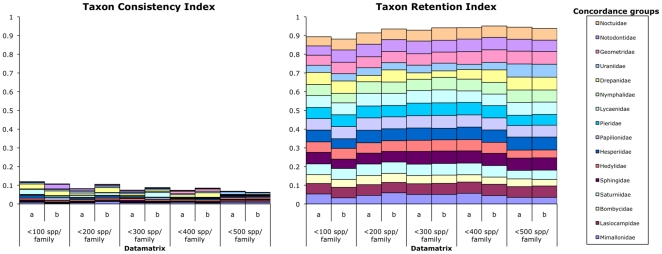
Phylogenetic signal scores. The bars are coloured to show the relative contribution to the score by each of the concordance groups.

This study provides an example of phylogenetic analysis using a datamatrix of short molecular sequences generally failing to promote the recovery of currently recognized families as monophyla. Drepanidae (only five species barcodes were available for this family), Lycaenidae and Notodontidae were recovered as monophyla at the lowest taxon sampling level (≤100 species per family) but no families were recovered as monophyla at the highest taxon sampling level (≤500 species per family). The average number of clades formed per family (S^t^) doubled from eight at the lowest taxon sampling level to 16 at the highest taxon sampling level, with the highest observed being 55 clades for 500 species of Noctuidae, the most species rich family of Lepidoptera [32]. The main pattern of increased taxon sampling failing to increase the number of monophyletic families was easily seen through the decrease in TCI scores from 0.12 at the lowest taxon sampling level to 0.06 at the highest taxon sampling level (Figure 3).

Increased taxon sampling did not appear to break long branches, as observed by the stationarity of the average p-distances within family datasets and the datamatrices as a whole across all levels of taxon sampling (Table S1). This suggests that increased taxon sampling was not having the effect of shortening the average branch length across the cladograms.

The CI score for the cladograms followed the usual pattern of lower values for datamatrices with more species [3] indicating additional homoplasy in a datamatrix as species were added. However, the increased homoplasy did not appear to be having a very negative effect on phylogenetic signal as the TRI scores did generally increase when the number of species per family was increased. The TRI accounts for the number of species in the datamatrix (Figure 1) so could be considered the more informative measure of the strength of phylogenetic signal. The fact the TRI generally increased, albeit very slightly, indicates more, or at least the same level of, cohesion of species from a family in the cladograms as the number of species included in the datamatrix increased and suggest substantial phylogenetic signal below the ‘family branch’, perhaps at the genus and tribe level.

### DNA barcodes versus other genes

If DNA barcodes are typical of other short molecular sequences, or even more generally, typical of datamatrices with a small character-taxon ratio this presents some important considerations for the assembly of future phylogenetic datamatrices. Currently COI is the only gene region for which taxon sampling to the level employed in this study is possible, but even for COI only a small fraction of lepidopteran species have been sampled. Sequence information to perform the same taxon sampling study with other genes is currently non-existent. However, similar studies on smaller scales suggest the phylogenetic signal in other gene datamatrices and even concatenated multi-gene datasets follow a similar pattern to that observed here in COI with increased taxon sampling [23]. The increased taxon sampling strategy requires that additional species can subdivide longer branches, a requirement highly dependent on tree shape. COI, due to its location and mode of inheritance as a mitochondrial gene, may be particularly sensitive to tree shape, especially at deeper levels, as it could be considered to evolve in a speciational rather than phyletic pattern [37]–[38]. The results from this study suggest interspecies divergences in COI for lepidopteran species are constrained around 10–14% (uncorrected p-distance; Table S1).

The Lepidoptera branch of the Tree of Life project (http://www.leptree.net) is sequencing 24 nuclear genes from an exemplar set of species with the aspiration of resolving deep nodes in the lepidopteran phylogeny. Recent publications by the team have included up to five gene regions, and initial studies generally recovered families as monophyletic [10], [30]. However other studies using the typical gene regions employed by Lepidoptera phylogeneticists (*wingless*, *elongation factor 1 alpha* and *period*), have had varying success [17], [20], [24], [29]. The inclusion of a few species as exemplars, perhaps necessary when sequencing a huge number of nucleotides, increases the *a priori* probability of taxonomically related species nesting together and provides a weaker test of phylogenetic signal and taxonomic hypotheses [39]–[40].

### What makes a good phylogeny?

This current study assesses the strength of phylogenetic signal through taxonomic congruence. This rests on the assumption that branches on the macrolepidopteran phylogeny leading to families (as currently recognized) are ‘real’ events in history and that the strength of phylogenetic signal in a datamatrix is directly related to presence of these branches in a cladogram. We can be reasonably confident (but never certain) in this assumption as lepidopteran families have been repeatedly inferred as natural groups by different researchers using different kinds of characters. However, as most phylogenetic studies use exemplars as representatives of much larger units many of the species included here have never before been subject to cladistic analysis. To put this into perspective, Regier et al. [30] used 66 exemplar species to represent the whole Macrolepidoptera in their recent analysis.

Increased taxon sampling in a phylogenetic analysis has clear advantages; the statistical power of an analysis is increased with larger datamatrices [40] and including the maximum possible number of species must ultimately improve the stability of a classification scheme over time [39]. This consequence has been demonstrated in Lepidoptera using morphological datamatrices and the Gelechioidea [41]–[42]. When species are used as representatives of much larger groups, whose monophyly has never been reliably established, the ‘reality’ of even larger groups inferred as monophyla is extremely questionable. A more comprehensive species sample, including heterogeneous representatives, is undoubtedly a better test of taxonomic hypotheses but the availability of species with a full character set always limits sampling [41].

While DNA barcodes alone were not sufficient to reconstruct monophyly of families, increased taxon sampling did increase phylogenetic signal by one measure (the TRI) suggesting substantial signal below the ‘family branch’. The continuing efforts to resolve the backbone of the lepidopteran phylogeny [31] together with the rapidly increasing number of lepidopteran species represented by molecular data, largely by virtue of the Barcode of Life initiative [34], presents a unique opportunity to elucidate the first species-complete phylogeny for a large species rich group. Such a tree would be an invaluable resource for applied phylogenetics and macroecology research [43]. This will require the development of analytical tools along the lines of supermatrix, supertree or constrained tree approaches [44] to connect the incredible diversity of the Lepidoptera - the leaves and twigs on the tree resolved through DNA barcodes - to the deep branches resolved through phylogenomics [9].

## Materials and Methods

### Taxon sampling

I mined BOLD (www.barcodinglife.org [45]) (which incorporates GenBank COI records not sequenced as barcodes *per se* and published independently of BOLD) for DNA barcodes of species from macrolepidopteran families with barcodes available for at least two species. A single barcode from each available species was included in a large dataset (Table S2) (Figure 2). Alignment was performed in BioEdit [46]. From this large dataset, datamatrices with five different sampling levels were created: (1) ≤100 spp/family, (2) ≤200 spp/family, (3) ≤300 spp/family, (4) ≤400 spp/family, and (5) ≤500 spp/family (Figure 2). Given that intrafamilial relationships within the Lepidoptera are largely unresolved, species were selected randomly from the large dataset to achieve these sampling levels, or for the families where the target could not be reached all available species were included (Figure 2). To account for the considerable variation expected among randomly selected datamatrices this procedure was undertaken twice [23], producing 10 datamatrices in total.

### Phylogenetic analysis

Aligned matrices were analyzed using the phenomenological method of maximum parsimony in TNT (new technology searches using the default section and ratchet options [47]). While some may question a decision not to include “evolutionary” analyses, declining to choose an optimality criterion *a priori* allows one to pick and choose preferred inferences *a posteriori* [15]. For the purpose of this study, I follow the view that global parsimony still represents the boldest test of homology [15], [21], [48] and monophyly of taxa [39] while avoiding the use of process models that can lead to incorrect inferences being well supported [49]. Mean p-distances within a family sample and across the entire data matrix were calculated in MEGA [50].

### Phylogenetic signal

Sixteen family groups were designated as concordance groups [3] for tests of phylogenetic signal through taxonomic congruence (Figure 1). Quantification was incorporated in the form of two measures: (1) the taxon consistency index and (2) the taxon retention index [3] (Figure 1). Values for these indices were obtained by constructing datamatrices of characters relating to group membership (i.e. 1 = member, 0 = non-member) and scoring these characters in PAUP [51] on the trees produced from the parsimony analysis of the molecular characters. The best possible score is 1 and higher values indicate the taxa are closer to monophyly. Character congruence was measured through the consistency index (CI) and retention index (RI) in PAUP.

## Supporting Information

Table S1 **Details of the sampling schemes and concordance groups used in the study, and the mean p-distances within each concordance group and the entire datamatix for each sample.**
(XLS)

Table S2 **List of taxa used in the study.**
(XLS)

## References

[1] Hebert PDN, Cywinska A, Ball SL, DeWard JR (2003). Biological identifications through DNA barcodes.. Proceedings of the Royal Society of London Series B-Biological Sciences.

[2] Floyd R, Wilson JJ, Hebert PDN, Foottit RG, Adler PH (2009). DNA barcodes and insect biodiversity.. Insect Biodiversity: Science and Society.

[3] Wilson JJ (2010). Assessing the value of DNA barcodes and other priority gene regions for molecular phylogenetics of Lepidoptera.. PLoS One.

[4] DeSalle R (2007). Phenetic and DNA taxonomy; a comment on Waugh.. BioEssays.

[5] Savolainen V, Cowan RS, Vogler AP, Roderick GK, Lane R (2005). Towards writing the encyclopaedia of life: an introduction to DNA barcoding.. Philosophical Transactions of the Royal Society B-Biological Sciences.

[6] Tautz D, Arctander P, Minelli A, Thomas RH, Vogler AP (2003). A plea for DNA taxonomy.. Trends in Ecology and Evolution.

[7] DeSalle R, Egan MG, Siddall M (2005). The unholy trinity: taxonomy, species delimitation and DNA barcoding.. Philosophical Transactions of the Royal Society B-Biological Sciences.

[8] Hajibabaei M, Singer GAC, Hebert PDN, Hickey DA (2007). DNA barcoding: how it complements taxonomy, molecular phylogenetics and population genetics.. Trends in Genetics.

[9] Wahlberg N, Wheat CW (2008). Genomic outposts serve the phylogenomic pioneers: Designing novel nuclear markers for genomic DNA extractions of Lepidoptera.. Systematic Biology.

[10] Regier JC, Cook CP, Mitter C, Hussey A (2008). A phylogenetic study of the 'bombycoid complex' (Lepidoptera) using five protein-coding nuclear genes, with comments on the problem of macrolepidopteran phylogeny.. Systematic Entomology.

[11] Pollock DD, Zwickl DJ, McGuire JA, Hillis DM (2002). Increased taxon sampling is advantageous for phylogenetic inference.. Systematic Biology.

[12] Hillis DM, Pollock DD, McGuire JA, Zwickl DJ (2003). Is sparse taxon sampling a problem for phylogenetic inference?. Systematic Biology.

[13] Hedtke SM, Townsend TM, Hillis DM (2006). Resolution of phylogenetic conflict in large data sets by increased taxon sampling.. Systematic Biology.

[14] Hillis DM (1998). Taxonomic sampling, phylogenetic accuracy, and investigator bias.. Systematic Biology.

[15] Brower AVZ, DeSalle R, Vogler A (1996). Gene trees, species trees, and systematics: A cladistic perspective.. Annual Review of Ecology and Systematics.

[16] Simon C, Frati F, Beckenbach A, Crespi B, Liu H (1994). Evolution, weighting, and phylogenetic utility of mitochondrial gene sequences and a compilation of conserved polymerase chain reaction primers.. Annals of the Entomological Society of America.

[17] Zwick A (2008). Molecular phylogeny of Anthelidae and other bombycoid taxa (Lepidoptera: Bombycoidea).. Systematic Entomology.

[18] Kallersjo M, Farris JS, Chase MW, Bremer B, Fay MF (1998). Simultaneous parsimony jackknife analysis of 2538 rbcL DNA sequences reveals support for major clades of green plants, land plants, seed plants and flowering plants.. Plant Systematics and Evolution.

[19] Yang ZH (1998). On the best evolutionary rate for phylogenetic analysis.. Systematic Biology.

[20] Mitchell A, Cho S, Regier JC, Mitter C, Poole RW (1997). Phylogenetic utility of elongation factor-1 alpha in Noctuoidea (Insecta: Lepidoptera): The limits of synonymous substitution.. Molecular Biology and Evolution.

[21] Purvis A, Quicke DLJ (1997). Building phylogenies: Are the big easy?. Trends in Ecology and Evolution.

[22] Feil EJ (2004). Small change: keeping pace with microevolution.. Nature Reviews Microbiology.

[23] Mitchell A, Mitter C, Regier JC (2000). More taxa or more characters revisited: Combining data from nuclear protein-encoding genes for phylogenetic analyses of Noctuoidea (Insecta: Lepidoptera).. Systematic Biology.

[24] Brower AV (2000). Phylogenetic relationships among the Nymphalidae (Lepidoptera) inferred from partial sequences of the wingless gene.. Proceedings of the Royal Society of London Series B-Biological Sciences.

[25] Monaghan MT, Inward DJG, Hunt T, Vogler AP (2007). A molecular phylogenetic analysis of the Scarabaeinae (dung beetles).. Molecular Phylogenetics and Evolution.

[26] Vane-Wright RI, Boggs CL, WB, PH (2003). Evidence and identity in butterfly systematics.. Butterflies: Ecology and Evolution Taking Flight.

[27] Kristensen NP, Scoble MJ, Karsholt O (2007). Lepidoptera phylogeny and systematics: the state of inventorying moth and butterfly diversity.. Zootaxa.

[28] Wahlberg N, Braby MF, Brower AVZ, de Jong R, Lee MM (2005). Synergistic effects of combining morphological and molecular data in resolving the phylogeny of butterflies and skippers.. Proceedings of the Royal Society B-Biological Sciences.

[29] Warren AD, Ogawa JR, Brower AVZ (2008). Phylogenetic relationships of subfamilies and circumscription of tribes in the family Hesperiidae (Lepidoptera: Hesperioidea).. Cladistics.

[30] Regier JC, Zwick A, Cummings MP, Kawahara AY, Cho S (2009). Toward reconstructing the evolution of advanced moths and butterflies (Lepidoptera: Ditrysia): an initial molecular study.. BMC Evolutionary Biology.

[31] Mutanen M, Wahlberg N, Kaila L (2010). Comprehensive gene and taxon coverage elucidates radiation patterns in moths and butterflies.. Proceedings of the Royal Society of London Series B-Biological Sciences.

[32] Pogue MG, Foottit RG, Adler PH (2009). Biodiversity of Lepidoptera.. Insect Biodiversity: Science and Society.

[33] Hajibabaei M, Janzen DH, Burns JM, Hallwachs W, Hebert PDN (2006). DNA barcodes distinguish species of tropical Lepidoptera.. Proceedings of the National Academy of Sciences of U S A.

[34] Hebert PDN, deWaard JR, Landry J-F (2009). DNA barcodes for 1/1000 of the animal kingdom.. Biology Letters.

[35] Franz NM (2005). On the lack of good scientific reasons for the growing phylogeny/classification gap.. Cladistics.

[36] Kawahara AY, Mignault AA, Regier JC, Kitching IJ, Mitter C (2009). Phylogeny and biogeography of hawkmoths (Lepidoptera: Sphingidae): evidence from five nuclear genes.. PLoS One.

[37] Bazin E, Glemin S, Galtier N (2006). Population size does not influence mitochondrial genetic diversity in animals.. Science.

[38] Salisbury BA (1999). Misinformative characters and phylogeny shape.. Systematic Biology.

[39] Goloboff PA, Catalano SA, Mirande JM, Szumik CA, Arias JS (2009). Phylogenetic analysis of 73060 taxa corroborates major eukaryotic groups.. Cladistics.

[40] Wortley AH, Scotland RW (2006). Determining the potential utility of datasets for phylogeny reconstruction.. Taxon.

[41] Kaila L (2004). Phylogeny of the superfamily Gelechioidea (Lepidoptera: Ditrysia): an exemplar approach.. Cladistics.

[42] Bucheli SR, Wenzel J (2005). Gelechloidea (Insecta: Lepidoptera) systematics: A reexamination using combined morphology and mitochondrial DNA data.. Molecular Phylogenetics and Evolution.

[43] Webb CO, Donaghue MJ (2005). Phylomatic: tree assembly for applied phylogenetics.. Molecular Ecology Notes.

[44] Kress WJ, Erickson DL, Swenson NG, Thompson J, Uriarte M (2010). Advances in the use of DNA barcodes to build a community phylogeny for tropical trees in a Puerto Rican forest dynamics plot.. PLoS One.

[45] Ratnasingham S, Hebert PDN (2007). BOLD: The Barcode of Life Data System Molecular Ecology Notes.

[46] Hall TA (1999). BioEdit: a user-friendly biological sequence alignment editor and analysis program for Windows 95/98/NT.. Nucleic Acids Symposium Series.

[47] Goloboff PA, Farris JS, Nixon KC (2008). TNT, a free program for phylogenetic analysis.. Cladistics.

[48] Phillips AJ (2006). Homology assessment and molecular sequence alignment.. Journal of Biomedical Informatics.

[49] Kolaczkowski B, Thornton JW (2004). Performance of maximum parsimony and likelihood phylogenetics when evolution is heterogeneous.. Nature.

[50] Tamura K Dudley J, Nei M, Kumar S (2007). MEGA4: Molecular Evolutionary Genetics Analysis (MEGA) Software Version 4.0.. Molecular Biology and Evolution.

[51] Swofford DL (1998). PAUP*: phylogenetic analysis using parsimony Version 4.0b2a..

[52] Fibiger M, Lafontaine JD (2005). A review of the higher classification of the Noctuoidea (Lepidoptera) with special reference to the Holarctic fauna.. Esperiana.

[53] Scoble MJ (1992). Lepidoptera: Form Function and Diversity..

